# Persistent Postural‐Perceptual Dizziness: A Practical Approach to Diagnosis and Patient Communication

**DOI:** 10.1111/ene.70494

**Published:** 2026-02-09

**Authors:** Hüseyin Nezih Özdemir, Jasmin Charlton, Elvira Cortese, Arianna Di Stadio, David Herdman, Diego Kaski

**Affiliations:** ^1^ SENSE Research Unit, Department of Clinical and Movement Neurosciences, UCL London UK; ^2^ Department of Neurology Ege University Medical School İzmir Türkiye; ^3^ Audiology Department, School of Speech‐Language Pathology and Audiology, Faculty of Medicine Universidad de Valparaiso Valparaíso Chile; ^4^ Department of Mental and Physical Health and Prevention University of Campania ‘Luigi Vanvitelli’ Naples Italy; ^5^ St George's University Hospitals NHS Foundation Trust London UK

**Keywords:** diagnosis, patient‐centered care, persistent postural perceptual dizziness, PPPD, vestibular

## Abstract

**Background:**

Despite clear diagnostic criteria established in 2017, delivering and explaining the diagnosis of persistent postural‐perceptual dizziness (PPPD) can be challenging.

**Methods:**

We outline a step‐by‐step approach to clearly explain the diagnosis, underlying mechanisms and treatment options to patients with PPPD, followed by a brief overview of current treatment approaches. This approach is adapted from the Cambridge‐Calgary Consultation Model and previous recommendations for functional neurological disorders but is mainly based on our expertise accrued over decades and input from patients with PPPD.

**Results:**

The practical clinical framework for the assessment and management of PPPD includes structured history‐taking, bedside examination and patient‐centred communication strategies aimed at improving diagnostic confidence and therapeutic engagement.

**Conclusion:**

Our experience indicates that the clinical approach used may shape patients' understanding, engagement and overall management trajectory in PPPD. Good communication is essential for the diagnosis and management of this condition.

## 1 Introduction

Persistent postural‐perceptual dizziness (PPPD) is a functional vestibular disorder [1]. The main symptoms are fluctuating sensations of non‐spinning vertigo and/or unsteadiness, present on most days, lasting longer than 3 months [1]. Exposure to complex visual environments like supermarkets, crowded settings and both active and passive motion can exacerbate these symptoms [1]. It is the most frequent cause of chronic dizziness and prevalence peaks in middle‐age [2]. PPPD can profoundly impair daily life and reduce quality of life [3].

The underlying pathophysiological mechanism of PPPD is thought to involve distorted processing of body motion leading to an overestimation of self‐motion and thus a mismatch between actual and perceived movement [4]. The vestibular symptoms likely arise from altered motion perception, inducing changes in relevant cerebral cortical activity and disrupting perceived postural control, typically triggered by episode(s) that impair or threaten balance [4, 5]. Neurotic personality traits, bodily hypervigilance, comorbid anxiety and sensory sensitivities (such as aversion to patterned stimuli or loud noises) common in individuals with PPPD are predisposing factors [3, 4, 6, 7].

Functional neurological disorder (FND) accounts for 30%–50% of general neurology outpatient clinics with a wide range of disabling manifestations, including movement disorders, impaired cognition and altered awareness [8, 9]. Despite its frequency and treatability, diagnosing FND and discussing the condition and treatment options with patients remain challenging for clinicians and may inadvertently harm patients through diagnostic delay, inappropriate treatment and perpetuating false illness beliefs [10, 11]. Among the various subtypes, PPPD stands out as one of the most common presentations of FND [12]. From the patient's point of view, many people with PPPD struggle to describe their dizziness and often feel misunderstood or dismissed by clinicians, which can exacerbate distress and hinder effective care [13, 14].

Although PPPD is listed under somatic symptom and related disorders in the diagnostic and statistical manual of mental disorders, fifth edition (DSM‐5), one might argue that it is not a psychiatric condition [15]. Indeed, such a categorisation can be misleading for patients and some clinicians. The adoption of the biopsychosocial model—rather than conceptualising FND as psychological or as a conversion disorder—has transformed the way clinicians approach these patients [16]. For functional seizures and functional movement disorders, various communication and management strategies have been proposed [17, 18]. The Committee for the Classification of Vestibular Disorders of the Bárány Society introduced diagnostic criteria for PPPD in 2017 [19]. These criteria established PPPD as a rule‐in diagnosis and paved the way for further research [19]. Despite these criteria, difficulties in recognising, explaining, and managing PPPD remain, due to its variable presentation, lack of clear positive signs on examination, strong psychological dimension, and lack of diagnostic biomarkers. To address these challenges, we propose a practical framework for PPPD assessment, including history‐taking, examination, and patient‐centred communication strategies.

## 2 Practical Approach to Diagnose PPPD

The diagnosis of PPPD relies entirely on clinical evaluation [19], making accurate history‐taking and a careful neurological examination essential. A structured and standardised approach to history‐taking—alongside targeted questions that address the diagnostic criteria—is crucial for establishing the diagnosis.

Clinicians usually review past medical records before seeing patients. Preparation for the consultation helps gather information about the patient and is believed to improve consultation quality [20, 21]. Previous studies have shown a female predominance in PPPD, with a mean age around 50 years [2]. However, our data show that PPPD is also the most common cause of dizziness among younger men (unlike other common vestibular conditions such as vestibular migraine), peaking around 45 years [22]. Sex and gender should not be used to ‘discard’ a diagnosis of PPPD. Similarly, whilst a history of psychiatric disorders such as depression and anxiety are known predisposing factors for PPPD, their absence should not prevent clinicians from considering the diagnosis, nor does their presence confirm it [5]. The key point is that demographic features and past medical history can offer supportive clues for diagnosis but should not be used in isolation to confirm or exclude PPPD.

Although the diagnostic criteria for PPPD are clearly defined, patients often have difficulty describing their vestibular symptoms clearly [1, 19]. Understanding the true symptom behind a patient's ‘I feel dizzy’ is important for making an accurate diagnosis in patients with chronic symptoms (where the examination is likely to be normal). To allow patients to describe their symptoms openly, we ask: ‘Can you describe your symptoms without using the word dizziness?’. Patients may respond in various ways, for example, describing non‐spinning vertigo as ‘It's like I’m swaying, even when I’m standing still’ or ‘I feel like I’m on a boat’; unsteadiness as ‘I feel like I might fall’ or ‘I feel off balance’; and lightheadedness as ‘I feel like I’m going to faint’ or ‘My head feels light’. Responses such as ‘My feet are wobbly’ or ‘I feel spaced out’ indicate mild dissociative experiences [1]. For patients who have difficulty describing their symptoms, you may need to offer some suggestions ‘is it like a merry‐go‐round?’, ‘is it like the brain is moving inside your head?’.

A typical day in the patient's life provides valuable information about symptom severity, daily functioning, avoidance of social or work situations, and additional insights into mood and cognitive status [20, 23]. A clinician may ask, ‘Can you describe a typical day for you?’. Fluctuation in the severity of symptoms is one of the diagnostic criteria for PPPD and clinicians might ask, ‘Are there days when you feel completely well or much worse than usual?’ to explore this [19]. Avoided situations can offer clues about potential triggers and clinicians should inquire about these as well. They may ask, ‘What brings your dizziness on or makes it worse?’ Typical examples of complex visual stimuli include crowded streets and supermarket aisles; for active and passive motion, walking and using an elevator; and for upright posture, getting out of bed or standing up from a chair [1]. If patients are unable to elaborate in response to this open‐ended question, it is then acceptable to ask close‐ended questions [24].

A sudden onset is not typical in PPPD; symptoms develop gradually [25]. However, when PPPD is triggered by an acute vestibular episode, such as vestibular neuritis or a cerebellar stroke, patients may refer to the onset of that episode as the beginning of their current symptoms, which can unintentionally mislead the clinician. It is important to distinguish the acute event from the subsequent development of PPPD to reach the correct diagnosis. A detailed history, including the description of dizziness mentioned above and the course of symptoms, is essential to separate and then appropriately relate these conditions.

Asking about a patient's ideas, concerns, and expectations is a standard part of the medical interview [24]. It helps clinicians understand the patient's beliefs about their condition, tailor the consultation, and lay the groundwork for effective explanations [24]. Related questions and the approach are discussed below in ‘Step 1: Preparing the Ground’. A clinician may also ask, ‘Are there any other symptoms you’d like to mention?’. This question is useful for both exploring differential diagnoses and identifying overlapping functional symptoms the patient may have [25]. In a waiting list survey of people with chronic dizziness, patients attributed an average of ten different symptoms to their condition, which was associated with greater dizziness related disability, meaning the person may be misinterpreting new or ongoing symptoms as signs there is still ‘something wrong’, even if those symptoms are unrelated [26]. Psychiatric comorbidities, daily life stressors and sleep disturbances can predispose to, perpetuate and exacerbate PPPD [27, 28]. It is preferable to wait for an appropriate moment to ask about psychiatric symptoms and personal life rather than rushing into them [6] as these may not be relevant in many cases. This can even be left for the second appointment to avoid harming the therapeutic relationship [29]. During the history‐taking, phrases like ‘How have you been feeling lately?’ or ‘Feeling stressed can sometimes make symptoms worse’ may be better options than directly asking, ‘Do you feel depressed or anxious?’. Clinicians should bear in mind that psychiatric comorbidities are not present in all patients with PPPD. It is advisable to avoid language that implies the symptoms are purely ‘psychological’ or ‘stress‐related’ [30]. The same applies to family and childhood history. These can predispose individuals to FNDs, but rushing into them can be counterproductive because many patients will have already been made to feel that it is ‘all in your head’ or ‘stress‐related’ [8, 20].

The mental state examination should be normal, although some patients complain of ‘brain fog’ and short‐term memory difficulties [5]. The patient's mood may be anxious and anxiety‐driven hypervigilance or changes in speech pattern can be noticeable [20]. The key part of the examination is the gait and posture. Maladaptation following the triggering event can lead to poor strategies for gait and posture [4]. During the Romberg test, stiffened leg and neck muscles may reflect increased alertness when visual input is removed [4]. We often ask patients, ‘If you had to rate your imbalance right now from 0 to 10 (where 0 is perfect and 10 so unsteady you might fall), what would it be?’—a question that can highlight perceptual dysfunction related to balance [4]. This can be closely linked to heightened vigilance toward balance, which can also be probed by asking patients if they ‘carefully monitor [their] balance’. It is worth noting that actual falls are uncommon in PPPD; if present, other causes rather than PPPD should be considered [1]. A stiff, cautious gait with shortened steps is typical but can also be normal [25].

Several studies have investigated objective findings in PPPD [5]. Studies using posturography have revealed overall greater sway in more posturally demanding conditions (e.g., standing on foam) compared with controls [31], and a stiffened ankle strategy with high‐frequency body sway (3.5–8 Hz) [32]. Another study introduced a perceived‐instability measure that showed a markedly higher reproduced‐to‐observed sway ratio in PPPD patients than in controls [33]. People with PPPD may also perceive their head tilt as larger than it actually is, even though their standard tests of vestibular and otolith function appear normal [34]. Although group‐level differences have been reported in research studies comparing patients with PPPD to healthy controls, clinical brain imaging in individual patients is typically normal and is not required for the diagnosis of PPPD [5].

## 3 I Suspect My Patient Has PPPD. What Do I Do Now?

Discussions around current treatment options for PPPD revolve around a multimodal approach, including vestibular rehabilitation therapy (VRT), cognitive behavioural therapy (CBT), and pharmacotherapy [30]. However, establishing a clear two‐way communication with the patient is a fundamental first step in the management of any type of FND [16, 35]. Here, we outline a step‐by‐step approach for clearly explaining the diagnosis, underlying mechanisms and treatment options to patients with PPPD, followed by a brief overview of current treatment options. This approach is adapted from the Cambridge‐Calgary Consultation Model and previous recommendations for FNDs [8, 20, 23, 24, 29, 36, 37], but is mainly based on our expertise accrued over decades and input from patients with PPPD. We believe that the same clinical framework can also be applied to other chronic dizziness syndromes, including Mal de Débarquement Syndrome, that fall within the spectrum of functional neurological disorders.

### 3.1 A Step‐By‐Step Approach for Discussing PPPD With a Patient

#### 3.1.1 Step 1. Preparing the Ground

As for any FND, before communicating the diagnosis to the patient, it is important to prepare the ground, beginning with ‘taking the problem seriously’. This isn't just about communication; it reflects good clinical practice and helps build trust from the outset. The clinician's attitude and language can convey this. For example, one might say, ‘I don’t think your symptoms are made up’, and acknowledge the impact of the condition by adding, ‘I can see how the dizziness is impacting your everyday life’. These statements can be offered during the consultation or before delivering the diagnosis [36, 37]. A review of healthcare professionals' views on patients with FND revealed some negative perceptions, such as describing them as ‘attention‐seeking’ or ‘faking’ [10]. We believe that setting aside such misconceptions is especially necessary for the effective management of patients with PPPD, where the journey to receiving a diagnosis can be very long [38].

A further important aspect to set the scene is to actively explore the patient's beliefs during history‐taking with an empathic approach. This helps the clinician shape the conversation and tailor explanations, since patients' knowledge and understanding of their condition can vary greatly [24, 30] and to progress with evidence‐based therapies, the clinician's belief about the underlying condition needs to marry that of the patient's. The question: ‘What do you think is wrong?’ can be a good starting point to explore patient beliefs. Along similar lines, patients with PPPD often worry about an undiagnosed disease, saying for example ‘Could it be multiple sclerosis?’ or ‘Could it be a brain tumour?’. Or a patient might be concerned about serious outcomes, asking, ‘What if I can’t take care of my children?’ or ‘What if I can’t go back to work?’. When discussing the diagnosis, the clinician should address these beliefs and expectations directly reflecting on each patient's specific worries and needs.

#### 3.1.2 Step 2. Delivering the Diagnosis

Delivering a clear diagnosis is important but goes far beyond simply naming the condition, such as saying, ‘The condition you have is persistent postural‐perceptual dizziness’. Whilst this is preferable to stating what the patient does not have (e.g., ‘You don’t have vestibular migraine or any other neurological disease that could explain your dizziness’), PPPD is simply a descriptive term (summarising the patient's symptoms but saying nothing about the actual cause or mechanism) so for patients to truly understand the condition, the clinician needs to spend some time describing the reason that the symptoms have arisen. This should be considered part of delivering the diagnosis but is also the first step in management (see step 4); education is important for engaging patients in their treatment [35]. Clinicians should be confident in the diagnosis and communicate it in a considerate and non‐judgmental way [36]. It is preferable to deliver the diagnosis at the end of the first visit. It is advisable not to list other neurological conditions the patient does not have unless the patient brings them up due to fear or concern or they have been relevant in the development of the PPPD (e.g., BPPV or vestibular migraine) [27].

Whilst PPPD is usually well accepted by patients [39], the terminology can be confusing. Some patients misinterpret the word ‘persistent’ as meaning that the problem is ‘permanent.’ Likewise, some misinterpret ‘postural’ to mean sitting up straight or avoiding slouching. This is important, since they may then not accept the diagnosis or it may inadvertently encourage them to focus on correcting their posture. As such, it is helpful to outline each word, particularly that ‘persistent’ means the symptoms have been present a long time, but it doesn’t mean it will never go away; and ‘postural’ refers to being ‘upright and moving’. In our view, ‘perceptual’ is a redundant word since to some extent all types of dizziness can be described as a ‘percept’, but this is an academic point.

#### 3.1.3 Step 3. Showing the Diagnostic Reasoning

**FIGURE 1:**
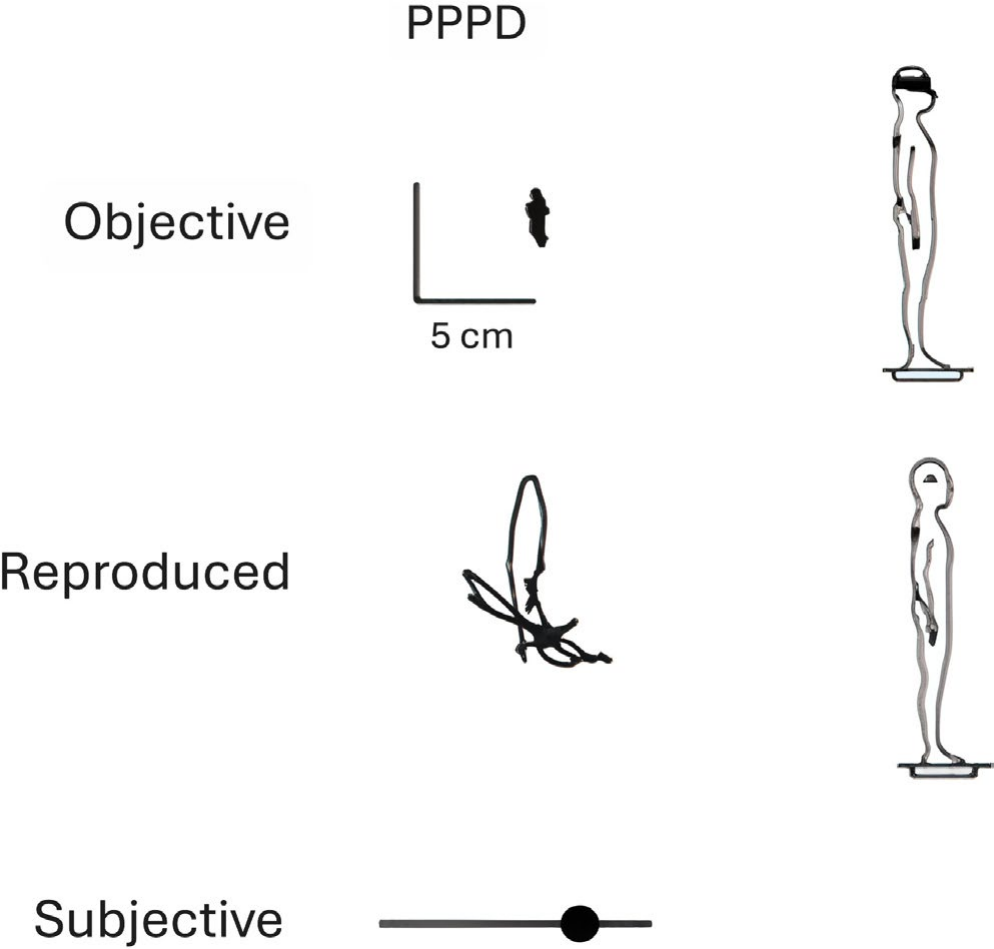
Centre of pressure horizontal trajectories over a single 20‐s posturography recording in a patient with PPPD: Observed sway with eyes closed (top) and ‘reproduced’ sway where the patient is asked to physically demonstrate how they felt they were moving when their eyes were closed (middle), and the patient's subjective rating of perceived instability on a visual analogue scale (0–10) (bottom). Note the marked difference between the two, which is characteristic of PPPD. The figure is modified and adapted from reference [33].

When explaining how the diagnosis was reached, as for other FND, focus on positive clinical features rather than emphasising normal findings or simply stating, ‘Your neurological examination is normal’ [23]. For people with PPPD, characteristic positive features include: dizziness that began following a triggering vestibular episode or an event that threatened or disturbed balance, the presence of symptoms occurring most days, worsening of symptoms in visually complex environments or busy settings, [25] and postural misperception (Video S1 and Figure 1) [40]. Providing a formal diagnosis with clear explanation can significantly benefit patients with FND [36]. In contrast, presenting the diagnosis solely through exclusion may inadvertently suggest to the patient that ‘there's no real problem’ [23].

#### 3.1.4 Step 4. Explaining the Mechanism

Explaining that PPPD is a functional neurological disorder, along with the mechanisms underlying it, can help patients gain insight into their condition [29]. In addition, providing information about the nature of functional disorders—specifically that they are not caused by visible abnormalities in the brain—can help patients understand why their MRI and other investigations are normal. Equally, it can be helpful to state that brain scans do not show abnormalities in how signals are being processed in the brain, analogous to computer software that is no less real or problematic than damage to the hardware.

Metaphors, albeit usually flawed, are useful for making complex medical concepts more accessible, but should be used with some caution since they can be misinterpreted so it is best to check the patient's own understanding afterwards. As above, we find it useful to compare the brain to a computer by saying ‘this is a software problem, not a hardware problem’ and explaining that it is a network disorder and that this is why there can be so many different symptoms [27, 29]. We also discuss how poor concentration and memory (‘brain fog’) can be a part of this network dysfunction.

In relation to the self—and visual motion—sensitivity that people with PPPD express, we find it useful to explain that this is due to the visual areas of the brain that process movement working ‘overtime.’ The state of vigilance that the brain is in (a protective mode when one senses danger) means that the brain is trying to sample sensory inputs excessively. In standing, healthy individuals sway, but we do not usually consciously attend to the sensation since our brain has learned that this is not a threat to our balance. In PPPD, akin to losing its ‘sensory filter’, it no longer knows what is relevant and what is not. This conversation can be directed to a discussion about the patient's heightened perception of their body movements. Demonstrating such postural hypersensitivity can be particularly helpful (Video S1) [23]. The clinician may demonstrate the patient's actual body movements during the eyes closed Romberg test by video‐recording this with the patient's consent and comparing this to how the patient perceives such movement (often excessively).

**FIGURE 2:**
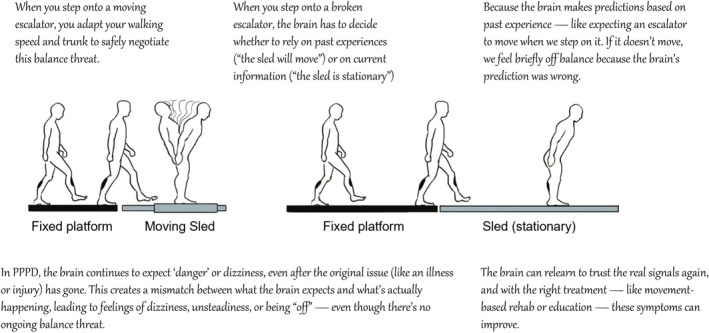
Using the ‘broken escalator phenomenon’ to explain how abnormal predictions of movement can result in persistent sensations of unsteadiness, in patients with PPPD.

Modern theories of perceptual disturbances, such as tinnitus, chronic pain, functional movement disorders and PPPD revolve around abnormal predictive coding [12]. Whilst these are complex neuroscientific concepts, they can be conveyed to patients in simpler terms to help them understand what is wrong. Thus, the brain is constantly predicting what's going to happen based on experience and then adjusting if the world doesn’t match those predictions. It's always asking: ‘What do I expect to sense right now?’ ‘What am I actually sensing?’ ‘How different are the two?’. When there's a mismatch, the brain can either update its prediction—or start relying more on its expectations than on real input. A simple analogy is that of the ‘broken escalator’ phenomenon (Figure 2), the strange feeling or jolt you experience when trying to step onto a broken escalator: ‘Your brain has a precise expectation towards motion since you've used escalators that usually move before and thus prepares your body for it (like subtle changes in gait speed and postural tilt) [40]. But when there is no motion, there is a mismatch between what your brain expected and what your body senses. This can be compelling, since it shows how very real physical symptoms can be experienced due to faulty predictions, rather than ‘bottom up’ structural damage’. The clinician can also use this example to provide hope, since the brain can ‘unlearn’ these patterns with practice.

When someone experiences a balance disturbance or threat—such as BPPV, concussion, presyncope, or an episode of vestibular migraine—the brain may start to doubt the reliability of sensory signals related to motion and posture. Over time, the brain can get stuck in this pattern, continuing to predict and ‘feel’ dizziness even after the original issue has resolved. A ‘better safe than sorry’ approach can persist, where the brain still interprets ambiguous or low‐level inputs (like minor swaying or visual motion) as important [41]. This mismatch between what the brain expects and what the body is actually experiencing can lead to persistent sensations of swaying or imbalance, driven more by faulty expectations than by current physical input. In this way, PPPD may be less about ongoing physical dysfunction and more about the brain's misfiring attempts to interpret and predict the body's state. You might say to the patient: ‘Your brain is designed to protect you. Sometimes it goes into a ‘better safe than sorry’ mode—it becomes extra alert to signals from your body in case something's wrong. After your balance system was disrupted, your brain kept scanning for danger. It's not that you’re imagining symptoms—it's that your brain is being overly cautious. This can lead to real sensations of dizziness or discomfort, even when your balance is okay’.

#### 3.1.5 Step 5. Reassuring That PPPD Is Common and Treatable

Emphasising that PPPD is common and treatable may prevent patients from seeing themselves as ‘a medical mystery’ and encourage them to engage in treatment [36]. A clinician might say, ‘I see many people with similar symptoms’, and ‘Whilst symptoms can be extremely disabling, it is not a sinister disorder causing ongoing damage or degeneration of the brain’. It is also important to empower the patient toward self‐help: ‘You can get better, but it's going to take some effort from you’. Cautious and realistic optimism without causing false hopes plays a supportive role in patient recovery [23].

For some patients, it can feel like they are being blamed for developing the disorder. Normalising responses to objectively challenging symptoms can help to reduce the stigma but open a new way of thinking from one of ‘illness’ to ‘recovery.’ A common and adaptive response to any aversive stimuli is avoidance, but continuous avoidance of activity appears to be a strong risk factor for developing persistent dizziness [42]. Therefore, what was once adaptive, such as avoiding moving quickly or consciously attending toward balance, has now become counter‐productive. The person is not to blame for this, but they can now start to take control of their recovery. Likewise, one can explain that almost all people, when exposed to a ‘postural threat’ such as walking on a high balance beam, will adopt certain movement patterns, such as walking slowly with smaller steps, reflective more of anxiety about falling, rather than their ability to perform the task. A simple demonstration in the clinic can help to explain this concept and the clinician might say, ‘your brain is behaving as if you’re always on that beam, even though you’re just walking on flat ground’.

#### 3.1.6 Step 6. Setting a Goal for the Treatment

Take the patient's expectations into account and encourage them to ask questions [23]. Uncovering what the patient is expecting from the consultation can guide the conversation toward ‘setting a goal’ for treatment, something particularly useful when managing persistent or functional symptoms. Clinicians might ask, ‘How do you hope to benefit from this visit?’ and ‘What can I do to help you?’. These questions can naturally lead into a statement like, ‘There is full potential for recovery; it just might take a bit more time and effort from you, but we will support you through this’. With this sentence, a clinician can emphasise that symptoms are reversible, treatment is a process rather than a quick fix, and the patient plays an active role in the treatment. We suggest clinicians answer any follow‐up questions in the same open and considerate tone. One should also take this opportunity to explore potential factors that can lead to a poor prognosis, such as ongoing negative life events, poor social support, severe mental health issues, particularly anxiety, a lack of acceptance of the disorder, or a strong feeling of injustice [43]. The clinician might ask whether there are any such factors at play: ‘for many people with these symptoms there may be factors that limit recovery. For example, ongoing stressors in life, severe anxiety, difficulty understanding the problem or even a strong feeling of injustice, of being ‘hard done by’. Do you think some of these may apply?’.

#### 3.1.7 Step 7. Shared Decision‐Making for the Treatment

When discussing the treatment options, it is important to involve patients in treatment decisions and emphasise their active participation in the process, as PPPD management should be individualised and ‘there is no magic pill’ that can instantly relieve symptoms [30]. Merely listing the treatment options would not be enough to help patients understand them. It is beneficial that clinicians explain the rationale behind the treatment options. A brief overview of the balance system can be helpful: ‘The brain relies on three inputs to maintain balance: visual input from the eyes, vestibular input from the inner ears and sensory input from the muscles and joints’. This leads to the explanation of visual dependency: ‘Your brain is currently relying too much on visual input’ [4]. The goal of treatment is to ‘retrain’ or ‘reprogram’ the brain to use all inputs more effectively. It can also be mentioned that medications are not central to treatment [34]. If relevant, patients should be advised to discontinue unnecessary vestibular suppressants [5]. It is also advisable that clinicians encourage patients to avoid over monitoring their symptoms.

#### 3.1.8 Step 8. Planning a Follow‐Up Visit and Closure

Planning a follow‐up visit helps prevent the patient from feeling dismissed and provides a safety net [23]. A statement such as ‘We’re not discharging you; you have a follow‐up appointment to check on progress’ can be reassuring. From the clinician's perspective, follow‐up visits offer an opportunity to explore predisposing and perpetuating factors, which may be difficult to address during the first consultation, as well as to monitor the patient's symptoms after treatment has begun [29]. Clinicians should be prepared for being asked more questions [29]. It is important that the clinician then work closely with the therapist(s), so as not to inadvertently disrupt their own therapeutic alliance. For example, ordering more investigations when a patient may be experiencing a normal and expected increase in symptoms as they start to engage in vestibular rehabilitation is likely to increase anxiety and diagnostic uncertainty, rather than be reassuring. Instead, encouragement to stick with treatment is usually the best approach, while reaching out to the therapist directly if there are concerns or suggestions.

Summarising the consultation and inviting final questions helps close the conversation clearly. To create a sense of safety, clinicians might say, ‘If you start feeling worse, don’t wait for the next appointment please contact us’. Ending with a question like ‘Is there anything you would like me to explain or go over differently?’ supports shared understanding [24]. In our experience, you will need time to explain and deliver the diagnosis to patients with PPPD, and this can be both difficult to manage in a general neurology setting, but also demotivating for clinicians who enjoy an “easy win”! One can choose not to engage with patients that have PPPD and refer them on to specialist clinics, but we feel that a ‘half‐way’ approach can be detrimental to patient care and often perpetuate perceptions that either there is a straightforward pharmacological or rehabilitation‐based approach (passive treatments) or that nothing can be done. Table 1 provides positive and negative examples for each step.

**TABLE 1:** Positive and negative examples for each step in discussing PPPD with a patient.

Step	Positive example	Negative example
1. Preparing the ground	‘Your symptoms are genuine’ ‘I don't think your symptoms are made up’ ‘I can see how the dizziness is impacting your everyday life’	Evaluating the patient as ‘attention‐seeking’ or ‘faking’
2. Delivering the diagnosis	‘The condition you have is persistent postural‐perceptual dizziness’	‘You don't have vestibular migraine or any other neurological disease’
3. Showing the diagnostic reasoning	‘Your symptoms follow the specific pattern we see in PPPD”	‘Your neurological exam is completely normal’
4. Explaining the mechanism	‘This is a software problem, not a hardware problem’ Demonstration of Romberg test with cognitive task	‘There is nothing neurological to explain the symptoms’
5. Reassuring that PPPD is common and treatable	‘I see many people with similar symptoms’ ‘It is not a sinister disorder’ ‘You can get better, but it's going to take some effort from you’	‘This is all in your head—just try to relax and it will go away.’ ‘There's no real treatment for this; you'll have to live with it.’
6. Setting a goal for the treatment	‘How do you hope to benefit from this visit?’ ‘What can I do to help you?’ ‘There is full potential for recovery; it just might take a bit of time and effort from you.’	Causing false hopes
7. Shared decision‐making for the treatment	‘The brain uses three inputs: vision, vestibular signals, and proprioception’. ‘Your brain is over‐relying on visual input’. ‘The goal of treatment is to retrain/reprogram the brain to use all inputs more effectively’.	Simply listing treatment options without explanation
8. Planning a follow‐up visit and closure	‘We’re not discharging you; you have a follow‐up appointment’. ‘If you feel worse, don't wait – please contact us’. ‘Does this all make sense?	Discharging the patient

### 3.2 A Practical Treatment Overview

The treatment options address the physical, psychological, and behavioural factors contributing to PPPD, as outlined by the biopsychosocial model [5]. Since there is no ‘one‐size‐fits‐all’ treatment, management should be tailored to each patient's specific needs [5]. There are three major treatment modalities for PPPD [44].

Vestibular rehabilitation therapy (VRT) aims to reduce visual dependency and recalibrate the integration of visual, vestibular, and proprioceptive inputs in the brain [30]. Although patients with PPPD often feel unsteady and fear falling, actual falls are uncommon [1]. This suggests that after the triggering event, their balance system continues to rely on ‘high‐risk’ strategies—such as increased dependence on visual and somatosensory input and a stiffened gait—rather than engaging vestibulo‐ocular and vestibulo‐spinal reflexes. VRT encompasses habituation exercises and postural stability training [45]. In this context, habituation refers to a reduction in dizziness due to repeated exposure to provocative stimuli—for example, repeatedly practicing head turns, bending and other head/body movements, or watching visual‐motion videos that trigger symptoms [45]. It typically begins with gentle exercises, with intensity gradually increased to avoid triggering excessive anxiety or fear responses to motion [1]. Although VRT is typically provided in person by physiotherapists, web‐based and virtual reality–based options have also been developed [30]. Patients with PPPD who have a longer duration of symptoms and multiple aggravating factors tend to benefit less from VRT [46].

Cognitive behavioural therapy (CBT) for PPPD typically includes patient education, identification of the patient's responses to dizziness, recognition of abnormal postural control, relaxation techniques, and exposure therapy. The specific components used, treatment duration and session frequency are highly individualised based on each patient's needs. Multiple studies have demonstrated CBT's efficacy in managing dizziness‐related symptoms. This intervention can be used as a standalone treatment, but we find that it is most effective if used in combination with VRT in the same sitting [30] an approach that we have termed ‘cognitive physical therapy’ [46]. This relies on physiotherapists being trained in CBT techniques, which is not widespread practice, but research is underway to address this.

Pharmacological treatment options for PPPD include serotonin reuptake inhibitors, either selective serotonin reuptake inhibitors (SSRIs) or serotonin‐norepinephrine reuptake inhibitors (SNRIs) [47]. No specific medication has been identified as the most effective and, in our experience, many patients are ‘sensitive’ to these drugs even at low doses [47]. Therefore, it is recommended to initiate treatment at lower doses than those typically used for depression [1, 37]. These medications can be used as adjuncts to other therapeutic interventions [5]. Antihistamines, benzodiazepines and betahistine are not recommended for PPPD [48].

Treatment selection should be guided by the patient's predominant symptoms, comorbidities, and psychosocial context [45]. For example, patients with marked visual dependence or motion sensitivity may benefit most from VRT focused on habituation, whereas those with significant anxiety, hypervigilance, or avoidance behaviours may require cognitive‐behavioural therapy to address maladaptive beliefs and fear‐driven movement patterns [45]. Patients with co‐existing mood symptoms may respond better when SSRIs or SNRIs are added [45].

The main limitation of this paper is that it is based primarily on clinical experience and expert opinion; therefore, its generalizability may be limited and future studies are needed to formally evaluate its impact on PPPD management and outcomes.

## 4 Conclusions

Persistent postural‐perceptual dizziness (PPPD) is among the most common causes of dizziness. We describe an approach to delivering the diagnosis derived from existing FND guidance. Our experience indicates that the clinical approach used may shape patients' understanding, engagement and overall management trajectory in PPPD. We hope that the guidance provided in this article will serve as a tool to improve patient outcomes.

## Author Contributions

**Hüseyin Nezih Özdemir:** Conceptualization, Data curation, Methodology, Project administration, validation, writing – initial writing, review and editng. **Jasmin Charlton:** validation, writing – review and editing. **Elvira Cortese:** validation, writing – review and editing. **Arianna Di Stadio:** validation, writing – review and editing. **David Herdman:** data curation, validation, writing – review and editing. **Diego Kaski:** conceptualization, data curation, methdology, validation, writing – review and editing.

## Funding

HNÖ is supported by the Turkish Neurological Society with an International Education Scholarship. DH is funded by the NIHR [Award ID: NIHR303566]. The views expressed are those of DH and not necessarily those of the NIHR or the Department of Health and Social Care. DK is supported by the National Institute for Health Research University College London Hospitals Biomedical Research Centre.

## Conflicts of Interest

The authors declare no conflicts of interest.

## Supporting information


**Video S1:** Example of a patient with PPPD. The video comprises three parts. In Part 1, the patient is asked to stand still with eyes open. In Part 2, the Romberg test is performed (eyes closed). In Part 3, the patient is asked to demonstrate their perceived body movements during the Romberg test. Notably, although there is no visible difference between the eyes‐open and eyes‐closed conditions (Parts 1 and 2), the patient demonstrates marked body rocking in Part 3, reflecting his subjective experience of imbalance.

## Data Availability

The data that support the findings of this study are available from the corresponding author upon reasonable request.
